# The German version of the Pulmonary Embolism Quality of Life (PEmb-QoL) questionnaire: reliability, responsiveness and structural validity

**DOI:** 10.1007/s11136-022-03120-3

**Published:** 2022-03-14

**Authors:** Simone Fischer, Christine Meisinger, Jakob Linseisen, Wolfgang von Scheidt, Thomas M. Berghaus, Inge Kirchberger

**Affiliations:** 1 Epidemiology, Faculty of Medicine, University of Augsburg, Stenglinstr. 2, 86156 Augsburg, Germany; 2 Clinical Epidemiology (KEPI), Helmholtz Zentrum München, Neuherberg, Germany; 3 Institute for medical Information Processing, Biometry and Epidemiology (IBE), LMU München, Munich, Germany; 4 Department of Cardiology, Respiratory Medicine and Intensive Care, University Hospital Augsburg, Augsburg, Germany

**Keywords:** PEmb-QoL, Pulmonary embolism, Questionnaire, Psychometric evaluation, Confirmatory factor analysis, Health-related quality of life

## Abstract

**Purpose:**

The Pulmonary Embolism Quality of Life (PEmb-QoL) questionnaire is the only existing disease-specific instrument for measuring quality of life after pulmonary embolism (PE). It includes six dimensions: frequency of complaints, limitations in activities of daily living, work-related problems, social limitations, intensity of complaints and emotional complaints. The present study aimed to determine the psychometric properties including responsiveness and structural validity of the German version.

**Methods:**

The analysis used data from participants of the LEA cohort study at University Hospital Augsburg. The PEmb-QoL was administered via postal surveys 3, 6 and 12 months post-PE. Internal consistency and test–retest reliability were evaluated by calculating Cronbach’s alpha and intra-class correlation coefficients (ICC). Standardized response means (SRM) were calculated for investigating responsiveness. For evaluating the fit of the factor structure, confirmatory factor analysis (CFA) was conducted.

**Results:**

Overall, we used data from 299 patients 3 months after PE. Cronbach’s alpha (0.87–0.97) and ICC (0.53–0.90) were in an acceptable to good range. SRM scores showed good responsiveness of all dimensions. CFA revealed the four-factor model including one general factor to have a good model fit.

**Conclusion:**

Despite existing floor effect, most standard criteria of reliability and validity were met and indications for appropriateness of the PEmb-QoL summary score could be found. Apart from some restrictions concerning the factor structure and the dimension of social limitations, our results support the use of the PEmb-QoL questionnaire for evaluating PE-specific quality of life. Future studies should seek replication in different samples to ensure generalizability of the findings.

**Supplementary Information:**

The online version contains supplementary material available at 10.1007/s11136-022-03120-3.

## Introduction

Pulmonary embolism (PE) describes an obstruction of the pulmonary arteries mostly originating from deep venous thrombosis of the leg or pelvic veins [1]. PE belongs to the most common acute cardiovascular diseases after myocardial infarction and stroke and incidence rates are increasing [2, 3]. Due to improved therapy and disease management, more patients survive an acute PE event [2]. Patients after PE can suffer from lasting symptoms, right heart failure and chronic thromboembolic pulmonary hypertension (CTEPH) [4]. While CTEPH is a severe but rather rare secondary disease [2], more than half of patients struggle with persistent or deteriorating dyspnoea and poor physical performance 6 months to 3 years after PE [5]. Some studies also report mental health problems such as anxiety disorders and depression after PE [6–8]. Since PE negatively affects different dimensions of health, it is important to consider health-related quality of life (HrQoL) as an outcome of PE health care. The disease-specific Pulmonary Embolism Quality of Life (PEmb-QoL) questionnaire was developed and validated in 2010 and covers six dimensions of HrQoL after PE with 40 items [9, 10]. After it was originally developed in Dutch and translated into English, it has subsequently been translated and validated in four different languages: Norwegian [11], Chinese [12], French [13] and German. The German version was translated by Frey et al. and it has been shown to meet standard psychometric criteria of reliability and validity [14, 15]. One study investigated the minimal clinically important difference (MCID) of the PEmb-QoL to be 15 units, which is important to assess relevance of observed changes [16]. Some other longitudinal studies, which did not primarily aim to examine the responsiveness of PEmb-QoL, are indicating its ability to detect change over time [17, 18].

Moreover, the structural validity of the PEmb-QoL questionnaire has not been comprehensively investigated. In the development and validation process of a questionnaire, explanatory factor analysis (EFA) is a commonly employed method and useful for discovering a set of unknown factors. To confirm a hypothesis about the number of underlying factors of the instrument, a confirmatory factor analysis (CFA) should be conducted [19, 20]. In addition, the dimensions of the original PEmb-QoL questionnaire were created based on the content of items and not based on EFA. The results of EFA in the first validation study of Klok et al. already showed slightly different results than the proposed factorial structure [9]. Other validation studies using EFA also reported different factor structure, e.g. three [13] or four [12] factors instead of the six original dimensions.

To the best of our knowledge, the PEmb-QoL questionnaire is the only existing and widely used disease-specific instrument for measuring HrQoL after PE; therefore, it is essential to comprehensively investigate its psychometric properties. Thus, the specific aim of the present study is to determine acceptability, reliability, responsiveness and structural validity using CFA of the German version of the PEmb-QoL questionnaire.

## Methods

### Sample and data collection

For the present study, data from the Lungenembolie Augsburg (LEA) study were used. The LEA study is a long-term observational cohort study including patients 18 years and older with PE who were treated at the University Hospital Augsburg. After discharge, the participants received postal questionnaires about PE-related topics including the PEmb-QoL questionnaire after 3, 6 and 12 months. Detailed information about the study design can be obtained from the published study protocol [21].

For the test–retest reliability, some of the participants were contacted by telephone to complete the items of the PEmb-QoL questionnaire again.

### Measures

#### PEmb-QoL questionnaire

The PEmb-QoL questionnaire is a disease-specific quality of life questionnaire and comprises 38 items in six dimensions: frequency of complaints, limitations in activities of daily living (ADL), work-related problems, social limitations, intensity of complaints and emotional complaints [9]. Two additional items are descriptive and do not belong to one of the dimensions. The items are answered on two-point to six-point Likert response scales referring to experiences during the past 4 weeks. Dimension scores were calculated by taking the mean of all items and were finally transformed into a scale from 0 to 100. Higher scores indicate worse quality of life. Since the developers of the PEmb-QoL questionnaire did not provide details on how to address missing data, we partially adopted an approach that Tavoly et al. already applied in the Norwegian validation study. If 50% or less of the data within a dimension were missing, the values were replaced by the mean value of the same dimension [11]. We used this approach for all our analyses except for the CFA. Similarly to some previous studies, we also calculated a summary score for overall PE-related quality of life by using the average of scores of all dimensions [13, 14, 16].

Since we had no global rating of change in the survey for analysing responsiveness of the PEmb-QoL questionnaire, the EQ-VAS and CRQ were used as external criteria instead.

#### EQ-VAS

The EQ-VAS is a visual analogue scale and part of the EQ-5D that is a validated measure of health status. The EQ-VAS ranges from 0 to 100 with higher scores indicating better subjective health status [22]. An MCID of around 7 in patients with chronic obstructive pulmonary disease has been reported previously [23].

#### CRQ

The Chronic Respiratory Disease Questionnaire (CRQ) includes 20 items across four domains: dyspnoea, fatigue, emotional function and mastery. Items are rated on a 7-point Likert response scale with higher scores indicating less impairment. The German version of the CRQ was developed and validated in 2004 [24]. Previous studies report the MCID to be an average per item change of 0.5 [25]. In the present study, only the dyspnoea dimension of the self-administration version was applied.

### Statistical analyses

Demographic and clinical characteristics were summarized as means and standard deviations or numbers and percentages. The distributions of the dimensions are described as means with standard deviations and medians with interquartile ranges.

For investigating acceptability, completeness of the data and floor and ceiling effects, which are presented as proportions of participants with minimal and maximal possible scores, were calculated. By adopting a rule of thumb proposed by Terwee et al., we considered them acceptable if they accounted for < 15% [26].

Internal consistency was measured by Cronbach’s alpha and average inter-item correlation. Internal consistency is a measure to test whether items measure the same underlying construct and was considered appropriate if Cronbach’s alpha ranges between 0.70 and 0.95 [26].

Test–retest reliability was assessed by contacting 56 participants who returned the questionnaires after 6 or 12 months by telephone to fill in the PEmb-QoL questionnaire again. A time interval of 10 to 20 days between test and retest was considered as appropriate to minimize potential memory effects but also to avoid the occurrence of a real clinical change in that time frame [26]. To ensure that no clinical change has occurred, participants were asked if their PE-related symptoms had changed in the last 2 weeks. Intra-class correlation coefficients (ICC) based on a two-way mixed effects model with absolute agreement were calculated and values > 0.75 were considered as good [27]. To examine the potential effect of the different data collection methods for conducting test and retest, we used the approach of generalizability theory [28] (see Supplementary Material).

For the present study, we considered responsiveness as the ability to detect change over time. First, we investigated the associations of PEmb-QoL with the external criteria EQ-VAS and CRQ by calculating Pearson correlation coefficients. For evaluating responsiveness, we divided the sample into three groups of participants whose health status (EQ-VAS) or dyspnoea (CRQ) improved, remained stable or deteriorated according to the MCID. For each group we calculated the standardized response mean (SRM) of the change of the PEmb-QoL scores for 3 and 6 months, 3 and 12 months and 6 and 12 months after PE. The SRM is an effect size calculated by dividing the mean change between two measurements by the standard deviation of the change score [29] and can be interpreted by Cohen’s rule of thumb, which states 0.2 for low, 0.5 for moderate and 0.8 for large effects [30]. If patients’ EQ-VAS score or CRQ dyspnoea score changed by at least the amount of MCID, we also expected a change in PEmb-QoL scores of at least moderate size and a low or no effect if EQ-VAS and CRQ scores remained stable.

CFA was conducted to investigate which factor structure fits the data. For handling non-normally distributed scores, we used robust maximum likelihood estimation and full information maximum likelihood method to account for missing data. Model fit was assessed through various global goodness-of-fit indices: chi-square test statistics (χ^2^), Tucker–Lewis Index (TLI), Comparative Fit Index (CFI), root mean square error of approximation (RMSEA) and standardized root mean square residual (SRMR). For good model fit, the chi-square test statistics should be non-significant and the ratio χ^2^/df < 2, TLI and CFI ≥ 0.95 (or at least ≥ 0.90) [31], RMSEA ≤ 0.05 (or at least ≤ 0.08) and SRMR ≤ 0.05 (or at least ≤ 0.10) [32]. Furthermore, an item was considered to load on the factor if the standardized factor loading was statistically significant (*p* value < 0.05) and ≥ 0.4. We used Akaike information criterion (AIC), Bayesian information criterion (BIC) and Satorra–Bentler scaled chi-squared difference test (Δχ^2^_scaled_) to compare the nested models [33]. Lower AIC and BIC indicate better model fit [32]. Additionally, we compared RMSEA confidence intervals to evaluate statistical significance as proposed in some previous studies [34, 35]. All analyses were conducted with the statistic software R version 4.1.1 [36] and R package ‘lavaan’ [37] for CFA.

## Results

### Patient's characteristics

A total of 305 patients returned the follow-up questionnaire 3 months after PE. For one patient, no baseline data were available and five patients did not complete the PEmb-QoL questionnaire at all and were omitted from the analysis. Demographic and clinical characteristics of the 299 included patients are presented in Table 1*.* The age ranged from 18 to 87 years with an average of 63 (± 14.7) years and 44.2% of the sample were women. Eight per cent already had at least one PE in the past. Fifty-four per cent of the patients had high PE-related risk of death according to the simplified Pulmonary Embolism Severity Index (sPESI).

**Table 1:** Patient baseline characteristics

Variable	*n* = 299^a^
Age (years)	63.0 (14.7)
Sex, Female	132 (44.2)
School education	
Main school (8 years)	118 (42)
Secondary school (9 years)	86 (30)
High school (≥ 12 years)	72 (25.4)
No graduation	2 (0.7)
Other	5 (1.8)
Missing	16
BMI (kg/m^2^)	29.4 (6.5)
Missing	12
History of PE	23 (8.4)
Missing	24
PE localisation, bilateral	208 (77.6)
Missing	31
sPESI^b^	
High risk (≥ 1 point)	133 (54.3)
Missing	54
Cancer	49 (17.8)
Missing	25

^a^Values are expressed in absolute numbers and percentages or means with standard deviations

^b^sPESI: simplified Pulmonary Embolism Severity Index that uses six clinical variables (age, history of cancer, chronic cardiopulmonary disease, pulsations, systolic blood pressure and arterial oxyhemoglobin saturation) to classify patients into high or low PE-related risk of death [38]

### Psychometric characteristics of the German version of the PEmb-QoL questionnaire

#### Acceptability

Of 299 patients, 243 (81.3%) had no missing items after mean replacement. All dimensions had floor effects, ranging from 7.0% for emotional complaints to 53.2% for social limitations (Table 2). Ceiling effects were small for all dimensions except for work-related problems. Table 3 shows means and medians of PEmb-QoL dimensions for all follow-up questionnaires at 3, 6 and 12 months. Distribution characteristics of the single items as well as boxplots and tables with missings and floor and ceiling effects of the data from 6 and 12 months after PE are provided in the Supplementary Material.

**Table 2:** Acceptability and internal consistency of the PEmb-QoL (3 months after PE)

Dimension	Number of items	Missings^a^ % (*n*)	Floor effects %	Ceiling effects %	Cronbach’s alpha	Average inter-item correlation
Frequency of complaints	8	7.0 (21)	30.8	0	0.87	0.49
ADL limitations	13	9.7 (29)	20.4	2.3	0.97	0.71
Work-related problems	4	7.0 (21)	43.5	32.1	0.94	0.80
Social limitations	1	4.7 (14)	53.2	2.0	–	–
Intensity of complaints	2	1.7 (5)	32.4	0	–	–
Emotional complaints	10	3.0 (9)	7.0	0.3	0.91	0.51
PEmb-QoL summary score	38	18.7 (56)	2.7	0	0.96	0.47

*ADL* activities of daily living

^a^Dimension was considered as missing if  > 50% of the items had missing values; otherwise, missing values were replaced by the mean value of the same dimension

**Table 3:** Mean [standard deviation (SD)] and median scores [interquartile range (IQR)] of PEmb-QoL dimensions, EQ-VAS and dyspnoea dimension of CRQ at 3, 6 and 12 months after PE

Dimensions	3 months (*n* = 299)		6 months (*n* = 257)		12 months (*n* = 196)	
	Mean (SD)	Median (IQR)	Mean (SD)	Median (IQR)	Mean (SD)	Median (IQR)
Frequency of complaints	16.7 (20.4)	9.4 (0.0–27.3)	16.1 (20.6)	9.4 (0.0–25.0)	16.1 (22.3)	9.4 (0.0–21.9)
ADL limitations	32.9 (30.9)	26.9 (3.8–57.6)	31.5 (30.4)	22.7 (4.2–50.0)	31.0 (30.8)	19.2 (3.8–54.5)
Work-related problems	44.2 (45.8)	25.0 (0.0–100.0)	39.9 (44.9)	0.0 (0.0–100.0)	35.0 (44.2)	0.0 (0.0–100.0)
Social limitations	20.4 (27.4)	0.0 (0.0–25.0)	18.5 (27.2)	0.0 (0.0–25.0)	16.7 (26.5)	0.0 (0.0–25.0)
Intensity of complaints	22.7 (22.7)	20.0 (0.0–40.2)	21.5 (22.4)	10.0 (0.0–37.5)	21.8 (24.6)	10.0 (0.0–40.0)
Emotional complaints	23.8 (19.6)	20.0 (8.0–36.0)	21.7 (19.5)	18.0 (6.0–32.0)	22.0 (19.5)	18.0 (6.0–34.0)
PEmb-QoL summary score	26.2 (24.1)	18.6 (4.2–47.3)	24.7 (24.1)	16.2 (3.4–43.0)	23.4 (25.1)	10.8 (2.9–46.4)
EQ-VAS	66.3 (20.6)	70 (50–85)	68.3 (20.1)	70 (55–85)	69.1 (20.3)	75 (60–85)
CRQ (dyspnoea)	5.6 (1.5)	6 (4.6–7)	5.7 (1.5)	6.4 (4.7–7)	5.6 (1.5)	6.3 (4.4–7)

Higher scores indicate worse quality of life

*ADL* activities of daily living

#### Reliability

Cronbach’s alpha for internal consistency ranged from 0.87 for frequency of complaints to 0.97 for limitations in ADL. Average inter-item correlation ranged from 0.47 to 0.80 (Table 2).

For test–retest reliability, 56 patients were contacted by telephone on average 14 days (mean = 13.5, SD = 2.7) after they returned their 6- or 12-month follow-up questionnaire. Six patients did not want to participate in the telephone interview. Three patients answered that their PE-related health status had changed in the last 2 weeks and were excluded for the test–retest analysis. Finally, data of 39 to 47 questionnaires depending on the dimension were included for calculating ICC (Table 4*)*. Intra-class correlation coefficients were high (0.76–0.90) for all dimensions except for social limitation (ICC = 0.53).

**Table 4:** Test–retest reliability

Dimension	*n*	ICC (95% CI)
Frequency of complaints	42	0.83 (0.70; 0.90)
ADL limitations	43	0.85 (0.65; 0.93)
Work-related problems	46	0.76 (0.60; 0.86)
Social limitations	46	0.53 (0.29; 0.71)
Intensity of complaints	46	0.89 (0.81; 0.94)
Emotional complaints	47	0.87 (0.70; 0.93)
PEmb-QoL summary score	39	0.90 (0.82; 0.95)

*ADL* activities of daily living, *ICC* intra-class correlation, two-way mixed model—single measures, *CI* confidence interval

#### Responsiveness

Pearson correlation coefficient of the PEmb-QoL summary score and EQ-VAS was *r* = − 0.68 (95%-CI: − 0.74; − 0.60) and − 0.85 (95%-CI: − 0.89; − 0.82) for CRQ, respectively. Forty-two per cent of the patients had an improved score on the EQ-VAS, 36% a deteriorated score and 22% remained stable in the interval of 3 to 6 months after PE. SRMs for the PEmb-QoL in each group for three time intervals are shown in Table 5. In the group with improved EQ-VAS scores, the SRMs ranged from 0.51 for frequency of complaints to 0.71 for PEmb-QoL summary score. In the group with deteriorated EQ-VAS scores, the SRM ranged from − 0.89 to − 0.48, and from − 0.13 to 0.03 in the stable group.

**Table 5:** Responsiveness of the PEmb-QoL scores with EQ-VAS as an external criterion

Dimension	Time interval	Improved^a^ EQ-VAS	Stable EQ-VAS	Deteriorated EQ-VAS
Frequency of complaints	3–6 months	0.51^b^	− 0.02	− 0.48
ADL limitations		0.66	− 0.02	− 0.89
Work-related problems		0.67	− 0.09	− 0.56
Social limitations		0.60	− 0.13	− 0.49
Intensity of complaints		0.60	− 0.09	− 0.50
Emotional complaints		0.64	0.03	− 0.62
PEmb-QoL summary score		0.71	− 0.12	− 0.82
Frequency of complaints	6–12 months	0.43	0.23	− 0.49
ADL limitations		0.69	− 0.06	− 0.78
Work-related problems		0.75	0.18	− 0.57
Social limitations		0.61	0.08	− 0.47
Intensity of complaints		0.55	0.12	− 0.67
Emotional complaints		0.50	0.01	− 0.59
PEmb-QoL summary score		0.78	0.11	− 0.70
Frequency of complaints	3–12 months	0.50	− 0.08	− 0.43
ADL limitations		0.79	− 0.08	− 0.83
Work-related problems		0.80	0.02	− 0.58
Social limitations		0.65	0.07	− 0.44
Intensity of complaints		0.64	− 0.16	− 0.54
Emotional complaints		0.54	0.03	− 0.52
PEmb-QoL summary score		0.35	− 0.30	− 0.35

*ADL* activities of daily living

^a^Improved: (Δ > 7); stable: (− 7 < Δ > 7); deteriorated: (Δ < − 7), where Δ is the difference of EQ-VAS scores of the given time interval and 7 is the MCID of the EQ-VAS

^b^SRM: Standardized response mean was calculated by dividing the mean change score of the PEmb-QoL by the standard deviation of the change score

Table 6 shows the SRMs for the PEmb-QoL in the groups of the dyspnoea dimension of CRQ for three time intervals. Thirty-six per cent of the patients had improved scores on the dyspnoea dimension of CRQ, 37% had deteriorated scores and 27% remained stable in the interval of 3 to 6 months after PE. In the group with improved dyspnoea scores, the SRM ranged from 0.90 for frequency of complaints to 1.52 for PEmb-QoL summary score. In the group with deteriorated dyspnoea scores, the SRM ranged from − 1.34 to − 0.72 and from − 0.21 to 0.04 in the stable group.

**Table 6:** Responsiveness of the PEmb-QoL scores with CRQ (dyspnoea) as an external criterion

Dimension	Time interval	Improved^a^ CRQ (dyspnoea)	Stable CRQ (dyspnoea)	Deteriorated CRQ (dyspnoea)
Frequency of complaints	3–6 months	0.90^b^	− 0.21	− 0.82
ADL limitations		1.30	− 0.20	− 1.20
Work-related problems		0.97	0.00	− 0.85
Social limitations		0.91	0.04	− 0.90
Intensity of complaints		1.12	− 0.06	− 0.89
Emotional complaints		1.04	− 0.03	− 0.72
PEmb-QoL summary score		1.52	− 0.21	− 1.34
Frequency of complaints	6–12 months	0.80	− 0.09	− 0.60
ADL limitations		1.19	0.28	− 1.33
Work-related problems		1.02	0.33	− 0.93
Social limitations		0.92	0.16	− 0.77
Intensity of complaints		0.90	0.08	− 0.95
Emotional complaints		0.87	0.02	− 0.74
PEmb-QoL summary score		1.18	0.20	− 1.13
Frequency of complaints	3–12 months	0.85	− 0.02	− 0.68
ADL limitations		1.18	− 0.07	− 1.18
Work-related problems		1.08	− 0.03	− 0.59
Social limitations		0.93	0.14	− 0.67
Intensity of complaints		1.00	0.00	− 0.83
Emotional complaints		0.84	0.08	− 0.75
PEmb-QoL summary score		0.59	− 0.33	− 0.34

ADL activities of daily living

^a^Improved: (Δ > 0.5); stable: (− 0.5 < Δ > 0.5); deteriorated: (Δ < − 0.5), where Δ is the difference of CRQ scores of the given time interval and 0.5 is the MCID of the CRQ

^b^SRM: Standardized response mean was calculated by dividing the mean change score of the PEmb-QoL by the standard deviation of the change score

#### Confirmatory factor analysis

**Fig. 1:**
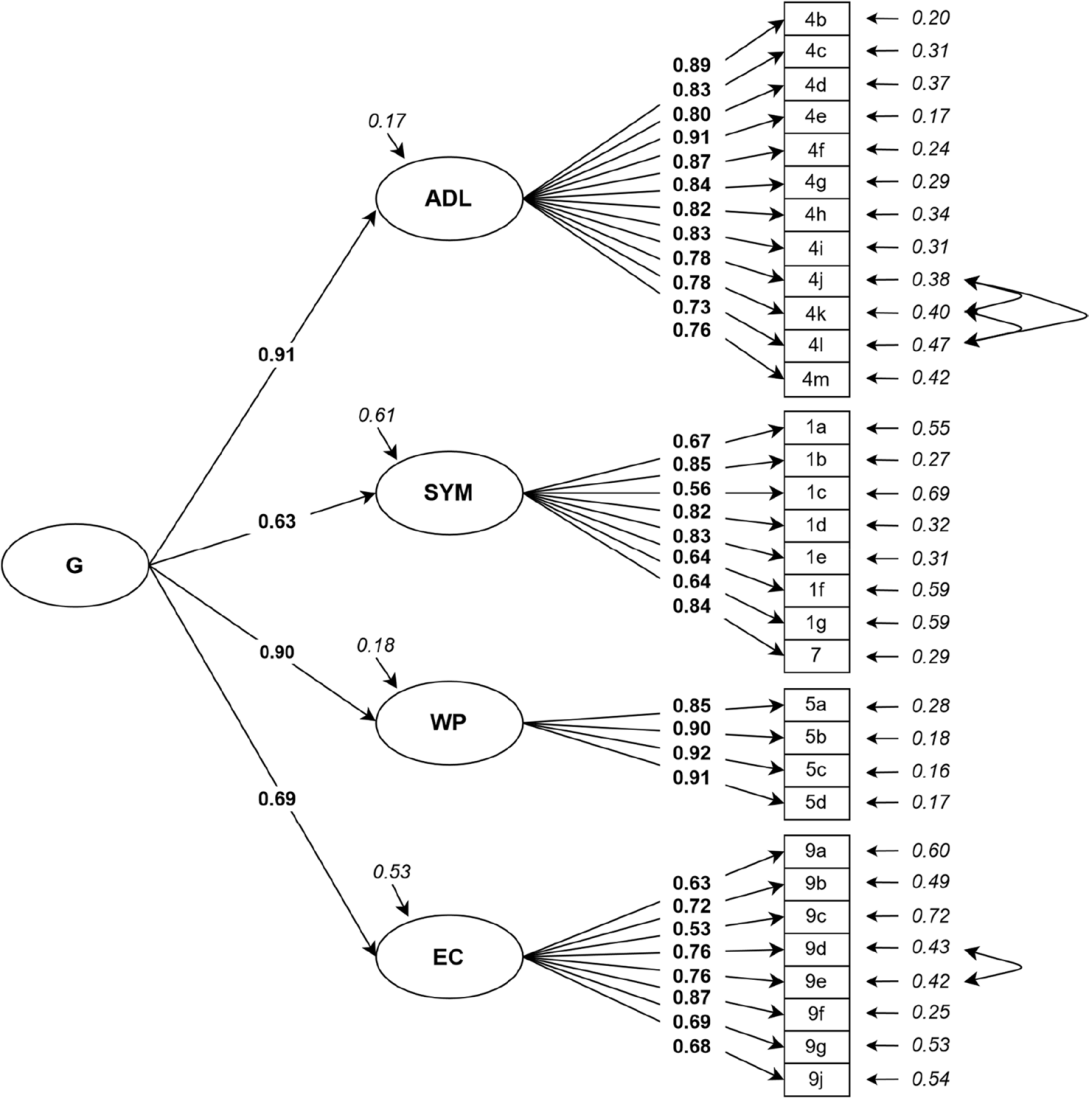
Standardized solution for model 5 (modified hierarchical model with four dimensions from Frey et al. and with one general factor); *G* General factor, *ADL* Activities of daily living, *SYM* Symptoms, *WP* Work-related problems, *EC* Emotional complaints. Factor loadings are in bold type, error variances are in italics, curved arrows represent co-varied error terms. All factor loadings were statistically significant with *p* < 0.001

We examined different models with CFA, which are explained in detail in the Supplementary Material. Fit indices of the models are shown in Table 7, according to which models 4 and 5 showed good global fit. The latent factor correlations between the four factors in model 4 ranged from 0.54 to 0.84; therefore, we added a general factor in model 5. Compared to model 4, the global fit indices of model 5 showed similar good fit with χ^2^/df = 1.85, *p* < 0.001, TLI = 0.928, CFI = 0.934, RMSEA = 0.060 (0.053; 0.066) and SRMR = 0.062. The factor loadings of all items on the four dimensions were still high, ranging from 0.53 to 0.92, as well as the factor loadings of the general factor (*Fig. **1*). We conducted a scaled chi-squared difference test for comparing nested models 3, 4 and 5. Model 4 is preferable over model 3 (Δχ^2^_scaled_ = 147.68, Δdf = 4, *p* < 0.001). Further, model 4 had better fit than model 5 according to the scaled chi-squared difference test (Δχ^2^_scaled_ = 15.693, Δdf = 2, *p* < 0.001). However, models 4 and 5 showed overlapping 90% RMSEA confidence intervals, suggesting a non-significant difference in fit between the models. Overall, the hierarchical model did not appear to provide a large decrement in model fit relative to model 4.

**Table 7:** Global fit measures for the MLR-estimates of the confirmatory factor analysis

	**Model 1** Six original dimensions	**Model 2** Five dimensions	**Model 3 ** Four dimensions (Frey et al.)	**Model 4** Four dimensions modified^d^	**Model 5** Four dimensions modified^d^ + general factor (hierarchical)
χ^2^	–^a^	1700.640	1055.422	825.213	842.523
df	–	620	458	454	456
χ^2^/df	–	2.74	2.30	1.82	1.85
*p* value (χ^2^)	–	< 0.001	< 0.001	< 0.001	< 0.001
TLI^b^	–	0.837	0.889	0.931	0.928
CFI^b^	–	0.848	0.897	0.936	0.934
RMSEA^b^ (90% CI^c^)	–	0.086 (0.081; 0.090)	0.074 (0.068; 0.080)	0.058 (0.052; 0.065)	0.060 (0.053; 0.066)
SRMR^b^	–	0.090	0.057	0.051	0.062
AIC	–	20,239.107	16,242.991	15,956.332	15,974.595
BIC	–	20,683.160	16,620.436	16,348.579	16,359.441

Estimator: robust maximum likelihood (MLR), *n* = 299, using full information maximum likelihood method

^a^For Model 1 covariance matrix of latent variables was not positive definite

^b^Robust fit indices

^c^Confidence interval

^d^Co-varied error terms on items 4j and 4k, 4k and 4l, 4j and 4l, 9d and 9e

## Discussion

Compared to the other German validation study by Frey et al., our sample was slightly larger, patients were younger with a higher proportion of women, and notably more patients had bilateral PE and cancer [14]. Except for sharing the same median for age, the same differences exist between our sample and the French validation study [13]. The Norwegian and the Chinese validation studies had notably younger patients included (mean age of 63 in comparison with 56 and 52) [11, 12]. The time between PE and study participation in the other studies differed considerably from our study. Frey’s study and the French validation study, for example, had a median time since PE occurrence of 15 months and the Norwegian study a median of 3.6 years [11, 13, 14].

In the present study, missings (more than 50% missing items in one dimension) were < 10% for all dimensions which indicates good acceptability. All dimensions except emotional complaints had substantial floor effects. Social limitations showed the highest floor effect with 53.2%, but it also comprises only a single question. Ceiling effects were observed in one dimension only: work-related problems. These results are in line with two other validation studies of the German version of the PEmb-QoL [14, 15]. Since floor effects may limit the ability to detect small changes, an analysis of the responsiveness of PEmb-QoL is crucial for the questionnaires’ applicability in long-term and intervention studies.

Internal consistency showed good to acceptable results. Cronbach’s alpha of limitations in ADL and work-related problems were almost higher than the recommended limit. This may be an indication for redundant items. The high average inter-item correlations of 0.71 and 0.80 for limitations in ADL and work-related problems also correspond with possible redundancy among items. Frey et al. found similarly high values for those two dimensions [14].

For the analysis of test–retest reliability, the time interval for the retest was on average 2 weeks. Since only three participants reported change, we assumed this interval to be appropriate for avoiding both memory effects and having a real change in PE-related health status. The ICCs were in a good range (> 0.75) except social limitations. It seems possible that the low ICC in social limitations may be related with the contact restrictions due to Covid-19 in Germany at the time of the retest and thus, respondents may have interpreted the question differently. Otherwise, it can be assumed that it is a problem of the wording of this question in the German version because Frey et al. also found low ICC for social limitations [14].

We investigated responsiveness of PEmb-QoL for the time intervals 3 to 6, 6 to 12 and 3 to 12 months. The size of the SRM was at least moderate for all dimensions and time intervals except for the PEmb-QoL summary score, which showed a lower SRM of 0.35 for the time interval of 3 to 12 months. The group that remained stable showed only low or no effects. As expected, the SRM was notably higher for the CRQ dyspnoea dimension than for EQ-VAS. The CRQ dyspnoea dimension includes questions about specific symptoms that are relevant for patients after PE, whereas EQ-VAS is only a global rating of subjective health status. Of interest, the SRM for the PEmb-QoL summary score was high for both EQ-VAS and CRQ, supporting the assumption that it may be suitable for representing overall PE-related quality of life. These results are supported by other studies that did not primarily examine responsiveness but used the PEmb-QoL questionnaire in a longitudinal study design. Kahn et al. used various HrQoL and health status questionnaires 1, 3, 6 and 12 months after PE. The results showed that the PEmb-QoL summary score is improving in alignment with improved scores of the Mental Component Summary score (MCS) and Physical Component Summary score (PCS) of the SF-36 and the University of California at San Diego Shortness of Breath Questionnaire (SOBQ) [17]. In addition, Chuang et al. reported mild to moderate effect sizes of the PEmb-QoL questionnaire 1 month after PE when a clinical event (e.g. bleeding, stroke, recurrent PE) has occurred in the meantime [18]. Together with results of test–retest reliability, our study contributes supportive information indicating the PEmb-QoL questionnaire to be an appropriate instrument for longitudinal studies. However, we did not have an objective external criterion for defining change, and responsiveness is a measure of one particular instrument applied to a particular sample and cannot be seen as absolute [39].

To our knowledge, this is the first study that uses CFA to evaluate the fit of the factor structure of the PEmb-QoL questionnaire. All models showed significant chi-square test statistics with *p* < 0.001, which would indicate bad fit. However, for some models, the ratio of χ^2^/df was < 2.0, which is being considered as good fit. It has also been discussed that models with robust estimation tend to be over-rejected by corrected chi-square test statistics [40].

While the four-factor structure showed good fit indices, the original six-factor structure could not be fitted to the data. We found very high correlations between intensity and frequency of complaints, which may suggest that the two dimensions are actually representing one factor for severity of symptoms. This assumption was already made in the very first validation study by Klok et al. [9]. For models 4 and 5, some re-specifications were applied. Co-varying of error terms should not be done just to improve model fit, but must be necessarily supported by theoretical rationale [41]. Since the re-specifications are theoretically reasonable and in each case within the same factor, we assumed them to be justifiable.

The model fit and high factor loadings of the hierarchical model (model 5) support that an overall summary score seems to be appropriate, but it has to be considered that in this model, some items (1h, 6, 8, 9h and 9i) of the original version are omitted. If the summary score should be able to compare the results of PEmb-QoL in different languages, changing the number of items should be treated with caution. Another aspect of the summary score is the potential loss of information about PE-related quality of life; as Tavoly et al. already pointed out, it is generally seen as a multidimensional construct [11]. Multidimensionality is supported by the structure of model 5, in which the general factor explains a high percentage (82–83%) of variance in the dimensions limitations in ADL and work-related problems, but for emotional complaints and symptoms about half of the variance is specifically explained by the dimension. Furthermore, it has to be considered that model 5 did not outperform model 4 as they showed similar fit indices and results of the scaled chi-squared difference test suggested model 4 to be the better model. However, even trivial differences may become significant and contrarily, overlapping RMSEA confidence intervals indicate no statistically significant difference. We assumed both models to show an adequate fit to the data, but model 5 may be favoured due to accounting for the high correlation between the four factors. Regarding the fact that by applying modifications, CFA loses its confirming character, the models should be validated in a different sample.

Since the current six existing dimensions in the questionnaire are debatable due to our and also previous study results, the often recommended PEmb-QoL summary score may be seen as a good option for interpreting and comparing results from different analyses. The two dimensions frequency and intensity of complaints could be considered to be interpreted as one dimension in the future analyses. Furthermore, the social limitations dimension should be interpreted with caution, because it includes only one question and psychometric properties did not show good results. Hence, practitioners should keep in mind to collect additional data about PE-related effects on social activity, if this is an information of interest. Additionally, if studies identify items not contributing to the measurement of the relevant concept, this may help developing short forms of the questionnaire, which are highly requested among clinical practitioners.

Our study has several limitations. While the respondents filled in the first questionnaire by themselves in written form at home, we conducted the retest by telephone. Therefore, our results for test–retest reliability are only comparable to a limited extent and have to be interpreted with caution. Furthermore, the sample may seem quite small for CFA due to the complex measurement model with 38 manifest variables. For assessing responsiveness, we were lacking a clinical assessment or judgement of medical experts as an external criterion for change of PE-related health status. Generalizability of our findings is also limited due to the fact that our sample comprises a cohort from a single university hospital in southern Germany. Patients admitted to university hospitals may differ from patients in non-university hospitals in terms of disease characteristics and treatment.

## Conclusion

In general, most standard criteria of reliability and validity were met and the results support the use of the German version of the PEmb-QoL questionnaire for evaluating PE-specific quality of life in longitudinal studies. However, we found high floor effects that should not be neglected when interpreting results from the questionnaire. Our study contributes knowledge about the responsiveness of the questionnaire and appropriateness of the PEmb-QoL summary score but also revealed some weaknesses of the German version. The construct validity regarding the original six-factor structure and especially the dimension social limitations are questionable. Factor structure has to be reconfirmed and responsiveness should be determined in comparison with other HrQoL measures or clinical criteria to create a comprehensive understanding for its ability to detect changes. Considering the limitations, our results should be seen as supportive data for reliability and validity and future studies should seek replication in different samples to ensure generalizability of the findings.

## Supplementary Information

Below is the link to the electronic supplementary material.

Supplementary file1 (DOCX 63 KB)

## Data Availability

Not available.

## Material availability

Not available.

## Code availability

Not available.
