# Supplementary material for: The German version of the Pulmonary Embolism Quality of Life (PEmb-QoL) questionnaire: reliability, responsiveness and structural validity
Source: Qual Life Res. 2022 Mar 14;31(7):2235–45. doi: 10.1007/s11136-022-03120-3 (PMC8919155; doi:10.1007/s11136-022-03120-3)
Supplement: Supplementary file 1 — Supplementary file1 (DOCX 63 KB) [file 11136_2022_3120_MOESM1_ESM.docx]

**Supplementary material:**

**The German version of the Pulmonary Embolism - Quality of Life (PEmb-QoL) questionnaire: reliability, responsiveness and structural validity.**

Simone Fischer^1,3^, Christine Meisinger^1,2^, Jakob Linseisen^1,2,3^, Wolfgang von Scheidt^4^, Thomas M. Berghaus^4^, Inge Kirchberger^1,2,3^

**Affiliations:**

1 Chair of Epidemiology, University of Augsburg, at University Hospital Augsburg, Augsburg, Germany

2 Clinical Epidemiology (KEPI), Helmholtz Zentrum München, Neuherberg, Germany

3 IBE, LMU München, Munich, Germany

4 Department of Cardiology, Respiratory Medicine and Intensive Care, University Hospital Augsburg, Augsburg, Germany

**Corresponding Author:**

Simone Fischer

IBE, LMU München

(c/o) Chair of Epidemiology, University of Augsburg

at University Hospital Augsburg

Stenglinstr. 2

86156 Augsburg, Germany

simone.fischer@med.uni-augsburg.de

**Distribution characteristics of single items of PEmb-QoL**

**Table 1:** Distribution of single Items (3 months after PE)

| **Item** | **Min** | **Q25** | **Median** | **Q75** | **Max** | **Missings** |
| --- | --- | --- | --- | --- | --- | --- |
| **1a** | 1 | 1 | 1 | 2 | 5 | 18 |
| **1b** | 1 | 1 | 1 | 2 | 5 | 19 |
| **1c** | 1 | 1 | 1 | 3 | 5 | 23 |
| **1d** | 1 | 1 | 1 | 2 | 5 | 19 |
| **1e** | 1 | 1 | 1 | 2 | 5 | 19 |
| **1f** | 1 | 1 | 1 | 1 | 5 | 20 |
| **1g** | 1 | 1 | 1 | 1 | 5 | 24 |
| **1h** | 1 | 1 | 2 | 4 | 5 | 7 |
| **4a** | 0 | 0 | 0 | 1 | 3 | 16 |
| **4b** | 1 | 1 | 2 | 2 | 3 | 36 |
| **4c** | 1 | 1 | 1 | 2 | 3 | 44 |
| **4d** | 1 | 1 | 2 | 3 | 3 | 36 |
| **4e** | 1 | 1 | 2 | 2 | 3 | 32 |
| **4f** | 1 | 1 | 2 | 2 | 3 | 28 |
| **4g** | 1 | 1 | 2 | 3 | 3 | 22 |
| **4h** | 1 | 1 | 1 | 2 | 3 | 28 |
| **4i** | 1 | 1 | 2 | 2 | 3 | 28 |
| **4j** | 1 | 1 | 1 | 2 | 3 | 39 |
| **4k** | 1 | 1 | 1 | 2 | 3 | 37 |
| **4l** | 1 | 1 | 1 | 2 | 3 | 31 |
| **4m** | 1 | 1 | 1 | 2 | 3 | 24 |
| **5a** | 0 | 0 | 0 | 1 | 1 | 22 |
| **5b** | 0 | 0 | 0 | 1 | 1 | 23 |
| **5c** | 0 | 0 | 0 | 1 | 1 | 20 |
| **5d** | 0 | 0 | 0 | 1 | 1 | 17 |
| **6** | 1 | 1 | 1 | 2 | 5 | 14 |
| **7** | 1 | 1 | 2 | 3 | 6 | 4 |
| **8** | 1 | 1 | 2 | 3 | 6 | 5 |
| **9a** | 1 | 1 | 2 | 3 | 6 | 7 |
| **9b** | 1 | 1 | 2 | 3 | 6 | 11 |
| **9c** | 1 | 2 | 3 | 4 | 6 | 18 |
| **9d** | 1 | 1 | 2 | 3 | 6 | 15 |
| **9e** | 1 | 1 | 1 | 3 | 6 | 25 |
| **9f** | 1 | 1 | 2 | 3 | 6 | 11 |
| **9g** | 1 | 1 | 1 | 2 | 6 | 9 |
| **9h** | 1 | 1 | 2 | 3 | 6 | 8 |
| **9i** | 1 | 1 | 2 | 3 | 6 | 9 |
| **9j** | 1 | 1 | 1 | 2 | 6 | 9 |

***Distribution of PEmb-QoL dimensions at 3, 6 and 12 months after PE***
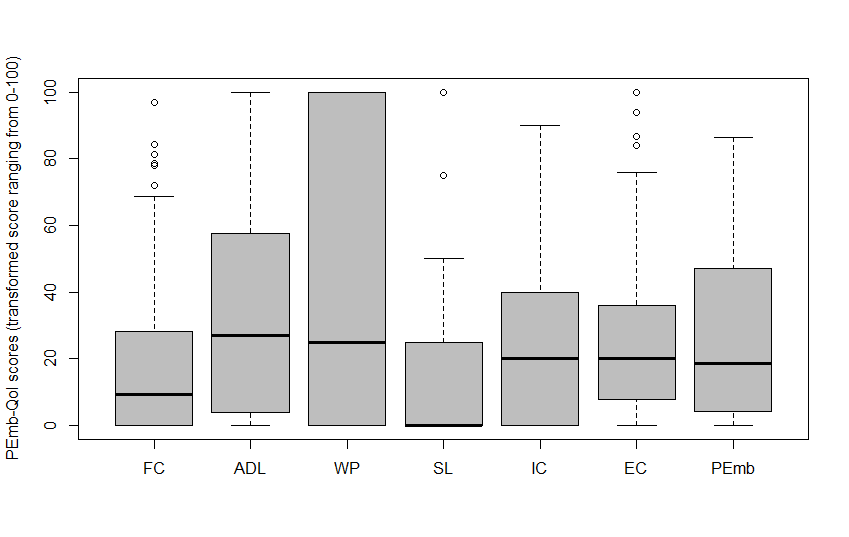


**Fig. 1:** Boxplots of PEmb-QoL dimensions and summary score (3 months after PE)

Higher scores indicate worse quality of life

FC: Frequency of complaints

ADL: Activities of daily living limitations

WP: Work-related problems

SL: Social limitations

IC: Intensity of complaints

EC: Emotional complaints

PEmb: PEmb-QoL summary score


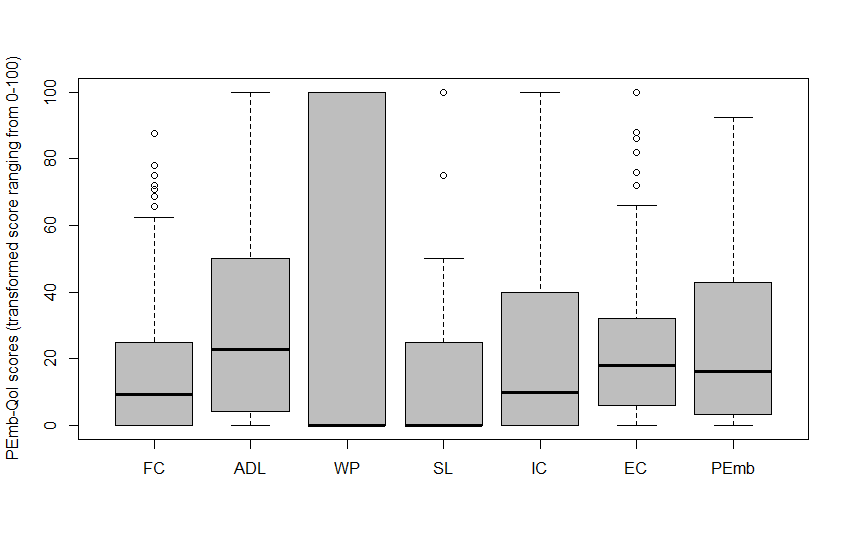


**Fig.2**: Boxplots of PEmb-QoL dimensions and summary score (6 months after PE)

Higher scores indicate worse quality of life

FC: Frequency of complaints

ADL: Activities of daily living limitations

WP: Work-related problems

SL: Social limitations

IC: Intensity of complaints

EC: Emotional complaints

PEmb: PEmb-QoL summary score


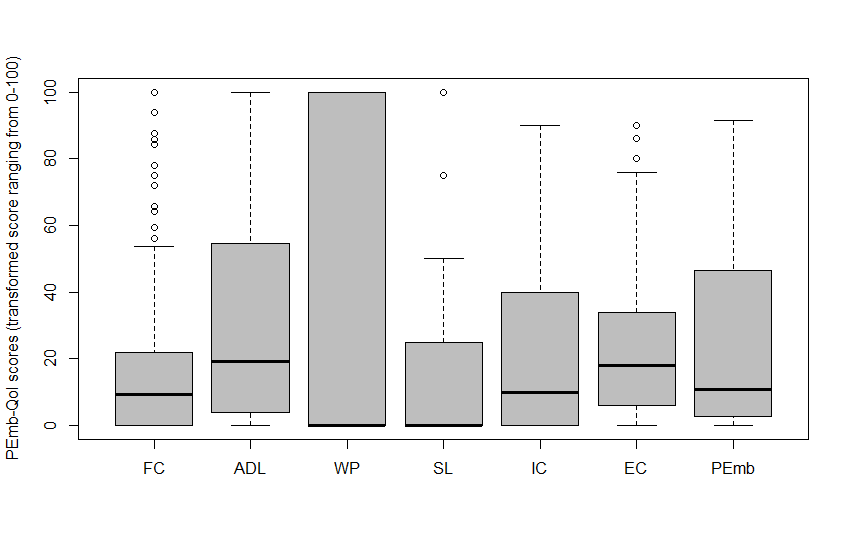


**Fig.3:** Boxplots of PEmb-QoL dimensions and summary score (12 months after PE)

Higher scores indicate worse quality of life

FC: Frequency of complaints

ADL: Activities of daily living limitations

WP: Work-related problems

SL: Social limitations

IC: Intensity of complaints

EC: Emotional complaints

PEmb: PEmb-QoL summary score

***Table 2:*** Missings, floor and ceiling effects of the PEmb-Qol (6 months after PE); n=257

| Dimension | Missings^a^  % (n) | Floor effects  % | Ceiling effects  % |
| --- | --- | --- | --- |
| Frequency of complaints (Q1) | 7.0 (18) | 32.0 | 0 |
| ADL limitations (Q4) | 6.2 (16) | 19.1 | 3.1 |
| Work-related problems (Q5) | 5.1 (13) | 49.0 | 28.0 |
| Social limitations (Q6) | 3.5 (9) | 57.6 | 2.3 |
| Intensity of complaints (Q7, Q8) | 1.2 (3) | 31.5 | 0.8 |
| Emotional complaints (Q9) | 1.2 (3) | 11.3 | 0.4 |
| Pemb-QoL summary score | 13.6 (35) | 4.7 | 0 |
| ADL: Activities of daily living  ^a^ dimension was considered as missing if >50% of the items had missing values, otherwise missing values were replaced with means | | | |

***Table 3:*** Missings, floor and ceiling effects of the PEmb-Qol (12 months after PE), n=196

| Dimension | Missings^a^  % (n) | Floor effects  % | Ceiling effects  % |
| --- | --- | --- | --- |
| Frequency of complaints (Q1) | 5.1(10) | 30.6 | 0.5 |
| ADL limitations (Q4) | 7.7 (15) | 23.0 | 2.0 |
| Work-related problems (Q5) | 4.1 (8) | 55.1 | 26.0 |
| Social limitations (Q6) | 3.6 (7) | 62.8 | 3.1 |
| Intensity of complaints (Q7, Q8) | 3.1 (6) | 36.7 | 0 |
| Emotional complaints (Q9) | 1.5 (3) | 11.7 | 0 |
| Pemb-QoL summary score | 12.8 (25) | 5.1 | 0 |
| ADL: Activities of daily living  ^a^ dimension was considered as missing if >50% of the items had missing values, otherwise missing values were replaced with means | | | |

**G-study according to the approach of generalizability theory with a single-facet design**

**Table 4:** Variance component results of the G-study

| *Source* | *df* | *SS* | *MS* | *Variance component* | *Percent*  *variance* |
| --- | --- | --- | --- | --- | --- |
| Frequency of complaints |  |  |  |  |  |
| person | 41.00 | 43553.08 | 1062.27 | 481.08 | 82.66 |
| method | 1.00 | 132.52 | 132.52 | 0.77 | 0.13 |
| error | 41.00 | 4104.91 | 100.12 | 100.12 | 17.20 |
| total | 48.76 |  |  | 581.97 | 100.00 |
| ADL  limitations |  |  |  |  |  |
| person | 42.00 | 64393.96 | 1533.19 | 720.48 | 85.44 |
| method | 1.00 | 1407.41 | 1407.41 | 30.59 | 3.63 |
| error | 42.00 | 3873.66 | 92.23 | 92.23 | 10.94 |
| total | 47.07 |  |  | 843.30 | 100.00 |
| Work-related problems |  |  |  |  |  |
| person | 45.00 | 151793.48 | 3373.19 | 1450.79 | 75.47 |
| method | 1.00 | 27.17 | 27.17 | 0.00 | 0.00 |
| error | 45.00 | 21222.83 | 471.62 | 471.62 | 24.53 |
| total | 57.34 |  |  | 1922.40 | 100.00 |
| Social limitations |  |  |  |  |  |
| person | 45.00 | 49565.22 | 1101.45 | 381.94 | 52.93 |
| method | 1.00 | 434.78 | 434.78 | 2.11 | 0.29 |
| error | 45.00 | 15190.22 | 337.56 | 337.56 | 46.78 |
| total | 70.29 |  |  | 721.62 | 100.00 |
| Intensity of complaints |  |  |  |  |  |
| person | 45.00 | 52530.43 | 1167.34 | 549.28 | 88.87 |
| method | 1.00 | 4.35 | 4.35 | 0.00 | 0.00 |
| error | 45.00 | 3095.65 | 68.79 | 68.79 | 11.13 |
| total | 50.29 |  |  | 618.07 | 100.00 |
| Emotional complaints |  |  |  |  |  |
| person | 46.00 | 36423.18 | 791.81 | 373.10 | 86.49 |
| method | 1.00 | 642.04 | 642.04 | 12.69 | 2.94 |
| error | 46.00 | 2098.14 | 45.61 | 45.61 | 10.57 |
| total | 51.63 |  |  | 431.40 | 100.00 |
| PEmb-QoL summary score |  |  |  |  |  |
| person | 38.00 | 38145.89 | 1003.84 | 475.93 | 89.97 |
| method | 1.00 | 93.15 | 93.15 | 1.06 | 0.20 |
| error | 38.00 | 1975.36 | 51.98 | 51.98 | 9.83 |
| total | 42.07 |  |  | 528.97 | 100.00 |
| ADL: Activities of daily living  df: degrees of freedom  SS: sum squares  MS: mean squares | | | | | |

Interpretation of results of the G-study:

We conducted a G-study according to the approach of generalizability theory with a single-facet design to examine the possible effect of the different data collection methods (paper and pencil at home vs. phone) on estimating reliability. The focus of generalizability theory is to identify multiple sources of measurement errors. Table 4 shows the measurement variance sources and the percentage of variance for every dimension. Data collection method shows low percentage of variance (0 - 3.6 %) and a large proportion of the variance is attributed to differences among persons in every dimension. Notable, the proportion of variance due to error is relatively high for dimensions work-related problems and social limitations (24.5 and 46.8 %). This may suggest problems with psychometric properties, which is in line with for example the lower ICCs that were calculated for these dimensions. However, variance explained by persons is higher than the error variance proportions for all dimensions. Results indicate that our different data collection methods seem to have only a small effect on estimating reliability.

**Selection of models for CFA:**

We conducted CFA with robust maximum likelihood estimation for five different models. Since 54.4 % of the participants indicated that they did not work, item 4a was excluded for the CFA. For the first model that represents the original six dimensions, the covariance matrix of latent variables was not positive definite and the model could not be analysed. Inspection of the correlation matrix led to model 2, in which the items of the two factors intensity and frequency of complaints were combined into one factor due to very high correlation. Model 2 showed poor model fit. Model 3 represents a model with four factors as proposed for the German version. The results of Frey et al. revealed a structure with four factors and some items that did not load well on any factor. They proposed to either delete the items or assign them to the dimensions they loaded the most. We examined both versions of the model (with and without the items in question), and the model without items 1h, 6, 8, 9h, and 9i showed better fit indices, which is why we decided for this model for further analyses. All of the error terms were uncorrelated and the latent factors were free to co-vary. Inspection of modification indices of model 3 revealed some local misspecifications, which we examined by analysing the wording of the questions. Items 4j, 4k and 4l do have the same wording except from different numbers of meters one can walk. Item 9d asks if the respondent felt more emotional and item 9e if it bothered the respondent that he or she felt more emotional, which makes the two items interdependent. As a result, we co-varied error terms on items 4j and 4k, 4k and 4l, 4j and 4l and 9d and 9e, which ended in model 4. To account for the high factor correlation between the four factors (0.54 to 0.84), we added a general factor in model 5. We assessed a bifactor model and a hierarchical (second order) model. The bifactor model also showed good fit indices (χ^2^/df = 1.76, p < 0.001, TLI = 0.936, CFI = 0.945, RMSEA = 0.056 (0.049; 0.063) and SRMR = 0.058). However, the bifactor model is less restricted than the hierarchical model 5 and therefore, is more likely to yield better fit statistics. After accounting for the general factor in the bifactor model some loadings on the domain specific factors were very low and not significant, which let us assume the hierarchical model 5 to be better in explaining the data.
